# Exosomal miR-100-5p inhibits osteogenesis of hBMSCs and angiogenesis of HUVECs by suppressing the BMPR2/Smad1/5/9 signalling pathway

**DOI:** 10.1186/s13287-021-02438-y

**Published:** 2021-07-13

**Authors:** Wu Yang, Weiwen Zhu, Yunfei Yang, Minkang Guo, Husun Qian, Weiqian Jiang, Yu Chen, Chengjie Lian, Zijie Xu, Haobo Bai, Tingmei Chen, Jian Zhang

**Affiliations:** 1 Department of Orthopedics, The First Affiliated Hospital of Chongqing Medical University, Chongqing, 400016 China; 2 Key Laboratory of Diagnostic Medicine Designated by the Ministry of Education, Chongqing Medical University, Chongqing, 400016 China; 3 Department of Geriatrics, The First Affiliated Hospital of Chongqing Medical University, Chongqing, 400016 China; 4 Department of Orthopedics, The First Affiliated Hospital of Sun Yat-sen University, Guangzhou, 510080 China

**Keywords:** Nontraumatic osteonecrosis of the femoral head, NONFH, Exosomes, Osteogenic differentiation, Adipocyte differentiation, Angiogenesis, miRNAs, BMPR2, miR-100-5p

## Abstract

**Background:**

Nontraumatic osteonecrosis of the femoral head (NONFH) is a common, progressive, and refractory orthopaedic disease. Decreased osteogenesis and angiogenesis are considered the main factors in the pathogenesis of NONFH. We aimed to figure out whether exosomes and exosomal miRNA from necrotic bone tissues of patients with NONFH are involved in the pathogenesis of NONFH and reveal the underlying mechanisms.

**Methods:**

RT-PCR and western blotting (WB) were used to detect the expression of osteogenic, adipogenic, and angiogenic markers. ALP staining and Alizarin Red S (ARS) staining were used to evaluate osteogenic differentiation of human bone marrow-derived mesenchymal stem cells (hBMSCs). Oil Red O staining was performed to assess the adipocyte deposition. A tube formation assay was used to study angiogenesis of human umbilical vascular endothelial cells (HUVECs). H&E staining and immunohistochemistry (IHC) staining were used to detect the effect of the NONFH exosomes in vivo. MicroRNA sequencing was conducted to identify potential regulators in the NONFH exosomes. The target relationship between miR-100-5p and BMPR2 was predicted and confirmed by a dual luciferase reporter assay and WB.

**Results:**

The NONFH exosomes reduced the osteogenic differentiation of hBMSCs and angiogenesis of HUVECs. In addition, the injection of the NONFH exosomes caused thinning and disruption of bone trabeculae in the femoral heads of rats. MiR-100-5p expression was upregulated in the NONFH exosomes and inhibited the osteogenesis of hBMSCs and angiogenesis of HUVECs by targeting BMPR2 and suppressing the BMPR2/SMAD1/5/9 signalling pathway. Silencing miR-100-5p expression rescued the reduction in osteogenesis and angiogenesis caused by the NONFH exosomes by activating the BMPR2/SMAD1/5/9 signalling pathway.

**Conclusion:**

The NONFH exosomal miR-100-5p can lead to NONFH-like damage by targeting BMPR2 and suppressing the BMPR2/SMAD1/5/9 signalling pathway, which may be involved in the pathophysiological mechanisms of nontraumatic osteonecrosis of the femoral head (NONFH).

**Supplementary Information:**

The online version contains supplementary material available at 10.1186/s13287-021-02438-y.

## Introduction

Nontraumatic osteonecrosis of the femoral head (NONFH) is a common, progressive, and refractory orthopaedic disease which usually results in substantial loss of function and inconvenience in the daily life of the patients [1–3]. Since the early diagnosis and treatment of NONFH is difficult and this disease is progressive, patients must eventually undergo hip arthroplasty [2, 3]. The difficulties in diagnosis and treatment for this disease mainly result from the unclear physiopathological mechanisms [4]. To date, the main hypotheses about the pathogenesis of SANFH include the imbalance between osteogenesis and adipogenesis of BMSCs [5] and the impairment of vessel endothelial cells (VECs) [6]. It was reported that in patients with NONFH, BMSC pools were damaged, and the osteoblasts are significant abnormal [7]. Vascular impairment is indicated by reduced circulating angiogenic cell function, with weakened migratory function and VEGF protein secretion [8]. Although BMSCs and VECs have strong proliferative potential, it is still difficult to reverse the disease progression of NONFH after pathogenic factors are removed. Therefore, we wondered whether necrotic bone tissues release some signals to impair the self-repair of BMSCs and VECs.

Exosomes are new mediators that participate in intercellular signal transmission [9]. Recently, these nanoparticles have been reported to be closely linked to many bone and joint diseases, including osteoarthritis, rotator cuff injury, and osteoporosis [10–13]. Exosomes were reported to influence osteogenic differentiation and angiogenesis to regulate bone reconstruction and homeostasis [14–17]. Recent studies have revealed that exosomes from multiple mesenchymal stem cells (MSCs) could be used for NONFH in rats [18–21]. However, no previous study has reported the effect of exosomes from necrotic bone tissues (NONFH exosomes) on the pathogenesis of NONFH. In addition, since cells in the tissue are surrounded by exosomes, BMSCs, and VECs treated with exosomes from necrotic bone tissue in femoral head are similar to BMSCs and VECs in necrotic regions. This phenomenon allows analysis of the physiopathological mechanisms of NONFH and identification of potential therapeutic targets for NONFH.

MicroRNAs (miRNAs/miRs), small noncoding RNAs, regulate many genes by binding 3′untranslated regions (3′UTRs) of their target mRNAs and ultimately cleaving or repressing translation of the mRNAs [22–25]. To date, some miRNAs have been reported to regulate NONFH by affecting proliferation and differentiation of BMSCs and VECs [26]. MiR-100-5p has been reported to be closely linked to some orthopaedic diseases, such as osteoporosis and osteoarthritis [27–30]. MiR-100-5p was also reported to be used for bone tissue engineering [31]. Wei et al. reported that miR-100-5p expression was notably upregulated in the blood of patients with NONFH [32]. However, no researchers have thoroughly explored the expression of miR-100-5p in NONFH exosomes and the role of miR-100-5p in NONFH.

In this study, we aimed to determine whether NONFH exosomes and exosomal miR-100-5p are involved in the pathogenesis of NONFH and to reveal the underlying molecular mechanisms. We hoped to reveal the detailed pathogenesis of NONFH and to provide therapeutic targets for the treatment of this condition.

## Materials and methods

### Patients and bone tissues

The study was conducted in accordance with the Declaration of Helsinki. All experiments were approved by the Research Ethics Committee of The Affiliated Hospital of Chongqing Medical University. Finally, 40 patients with NONFH (Acro stage III and IV) and 40 FNF patients who underwent hip arthroplasty at the First Affiliated Hospital of Chongqing Medical University from December 2018 to October 2020 were recruited. The femoral neck fracture (FNF) patients were considered the control group. The demographic data of the study groups are shown in Table 1. All of these femoral head samples were collected after they were resected and immediately divided into two halves with a bone knife. Part of the femoral head was rapidly placed in the liquid nitrogen for the next experiments, while the other part of each sample was fixed in 4% paraformaldehyde for histological analysis.

**Table 1:** Demographics data of the study groups

Group	Gender (male/female)	Side right/left	Age (years)	BMI (kg/m^**2**^)	Acro stage	Yield of exosomes (10^**10**^/g)
NONFH	24/16	21/19	63.95 ± 6.11	22.84 ± 2.71	stage III n = 19 stage IV n = 21	2.89 ± 1.15
FNF	22/18	16/24	64.08 ± 5.53	22.81 ± 2.01		1.26 ± 0.46

Note: Data are presented as mean ± standard error (SEM). “NONFH” represents the group of nontraumatic osteonecrosis of the femoral head. “FNF” represents the group of femoral neck fracture. “BMI” means body mass index

### Extraction of exosomes from bone tissues of the patients with NONFH and FNF bone tissues

Based on the previously reported methods, exosomes were extracted after the bone tissues were ground into homogenate with liquid nitrogen [33]. Isolation and purification were performed with a multistep ultracentrifugation process. In brief, the homogenate was centrifuged at 300*g* for 10 min, 1500*g* for 10 min, and 10,000*g* for 30 min. Next, the supernatant was centrifuged at 100,000*g* for 2 × 70 min. The centrifugal was performed at 4 °C. After every centrifugation, the supernatant was transferred to a new centrifuge tube. The solution without exosomes was also collected. Finally, the exosomes were washed by PBS and filtered with a 0.22-μm filter, and then stored in 100 μL PBS at − 80 °C. Some exosome pellets were lysed in RIPA and PMSF lysis buffer (RIPA:PMSF = 100:1) to extract proteins and detect the total protein concentration of the exosomes. The extracted proteins were stored at − 80 °C for WB.

### Characterization of exosomes

The size distribution of the FNF exosomes and the NONFH exosomes was measured by nanoparticle tracking analysis (NTA) using a NanoFCM N30E particle size analyser. The NTA data were analysed using the Zeta View software. The morphology of the FNF exosomes and the NONFH exosomes were visualized by a Hitachi HT-7700 transmission electron microscope (TEM). The exosomal biomarkers CD9, CD63, Alix, and TSG101 were analysed by western blotting. The calnexin also was selected as a positive control. The ultra-centrifuged supernatant mentioned above was also used as negative control in western blotting.

### Cell culture and transfection

Human bone marrow mesenchymal stem cells (hBMSCs), human embryonic kidney (HEK)-293 T cells, and human umbilical vein endothelial cells (HUVECs) were purchased from Otwo Biotech (Guangdong, China). Cells were cultured in Dulbecco’s modified Eagle’s medium (DMEM)/high glucose (HyClone, China) supplemented with 10% fetal bovine serum (Gibco, UK), 100 U/mL penicillin, and 100 μg/mL streptomycin (NCM, China) at 37 °C and 5% CO_2_. The medium was changed every 3 days. Both concentration of FNF exosomes and NONFH exosomes added into cells were 60 μg/mL medium (the concentration of the exosomes was detected by a BCA protein concentration detection kit).

The negative control (NC), agomiR-100-5p, antagomir-100-5p,siBMPR2, wild-type BMPR2, and mutant type BMPR2 plasmids (Genpharma, Shanghai, China) were transfected into cells with Endofectin^TM^-MAX (GeneCopoeia, USA) according to the manufacturer’s guidelines when cell confluence reached 50%. The working concentration of agomiR-100-5p was 100 nM, and those of antagomiR-100-5p, NC, and siBMPR2 were 200 nM.

### Cell uptake of exosomes

The exosomes were stained with a PKH67 kit (BestBio, China) according to the manufacturer’s instructions. The labeled exosomes were filtered using a 0.22-μm filter and dissolved in the sterile PBS. HBMSCs and HUVECs were treated by exosomes labeled by PKH67 and cultured in serum-free medium for 24 h. Then, the cells were washed with PBS and subsequently fixed with 4% paraformaldehyde for 30 min. Afterwards, the nuclei were stained by with 4′,6-diamidino-2-phenylindole (DAPI) for 10 min at room temperature and next the redundant dye was washed off. At last, the cells were observed and imaged under a fluorescence microscope (Leica, UK).

### Osteogenic differentiation, alkaline phosphatase staining, and Alizarin Red S staining

The hBMSCs were inoculated in 24-well plates. When cell confluence reached 80%, the medium was changed to osteogenic differentiation medium (Cyagen, USA). The medium was changed every 3 days and when the medium was changed, exosomes were added to the medium. Alkaline phosphatase (ALP) staining and Alizarin Red S staining were done to evaluate the level of osteogenic differentiation. After the hBMSCs were cultured in osteogenic differentiation medium for 7 days, ALP staining was conducted according to the manufacturer’s instructions (Beyotime, China). The cells were incubated with 10 mM p-nitrophenyl phosphate (Meilunbio, China) as the substrate at 37 °C for 15 min. Afterward, ALP activity was quantified at 420 nm by a microplate reader [34]. After cultured in osteogenic differentiation medium for 3 weeks, the hBMSCs were fixed, stained, and cleared according to the instructions of the Alizarin Red S staining kit (Solarbio, China). Based on the previously reported methods, the stained mineralization nodules were dissolved with 10% cetylpyridinium chloride at 37 °C for 30 min [35]. The solution was added to a 96-well plate, and a microplate reader was used detect the OD value at 562 nm for quantitative analysis.

### Adipocyte differentiation and Oil Red O staining

When cell confluence reached 100%, the medium was changed to the adipocyte differentiation medium (Cyagen, USA). The medium was changed every 3 days, and when the medium was changed, exosomes were added to the medium. After cultured in adipocyte differentiation medium for 4 weeks, the hBMSCs were stained with Oil Red O staining reagent according to the manufacturer’s instructions (Solarbio, China). Based on the previously reported methods, the stained lipid deposits were extracted with isopropyl alcohol at room temperature for 30 min [36]. The solution was added to a 96-well plate, and the OD value was measured at 510 nm for quantitative analysis.

### Wound healing assay

Cell migration of hBMSCs and HUVECs were detected by a wound healing assay. The cells were inoculated in six-well plates with the concentration of 8 × 10^5^ cells/well and treated with PBS, FNF exosomes, and NONFH exosomes. When the confluence was greater than 95–100%, the monolayer was scratched using a 10-μL sterile pipette tip and then cultured with the sterile serum-free medium supplemented with 60 μg/mL medium. The wound widths of the HUVECs and hBMSCs were observed using an inverted optical microscope (Leica, Germany) and analysed by ImageJ software.

### Tube formation assay

After treatment with PBS, FNF exosomes, NONFH exosomes, NC, agomiR-100-5p, antagomiR-100-5p, and siBMPR2 for 3 days, HUVECs were digested and seeded at 40,000 cells/well in Matrigel-coated 48-well plates. The cells were cultured in FBS-free medium supplemented with exosomes (50 μg/mL). Six hours later, tube formation was observed under an optical microscope. Tube-forming structures were analysed using ImageJ software to detect the tube length.

### RNA extraction

Total RNA was extracted according to the operation manual of Simply P Total RNA Extraction Kit (BioFlux, China). MiRNA was extracted according to the operation manual of Biospin miRNA Extraction kit (BioFlux, China). Finally, a spectrophotometer (Biodrop Ulite, UK) was used to detect the RNA concentration.

### MiRNA sequencing

High-throughput sequencing was performed by Novogene (Beijing, China). Briefly, Sequencing libraries were generated using NEBNext® Multiplex Small RNA Library Prep Set for Illumina® (NEB, USA), and index codes were added to the sequences of attributes of each sample following the manufacturer’s recommendations. The clustering of the index-coded samples was performed on a cBot Cluster Generation System using TruSeq SR Cluster Kit v3-cBot-HS (Illumina) according to the manufacturer’s instructions. Then, the Illumina HiSeq 2500/2000 platform was used to perform single-ended 50 bp sequencing of the library. Differential expression analysis of two groups was performed using the DESeq R package (1.8.3). T with P value ≥ 0.05. The miRanda database was used to predict the target genes of the differentially expressed miRNAs (DEmiRNAs). Cluster Profiler R software was used for Gene Ontology (GO) enrichment of the target genes, and KOBAS software was used for KEGG enrichment of the target genes.

### Real-time quantitative PCR

The mRNAs were reverse transcripted into cDNA using the PrimeScriptTM Reagent Kit with gDNA Eraser (TaKaRa, Japan). A 20 μL system was used for real-time quantitative PCR reaction, and 4 secondary pores were set. The cDNA gene amplification conditions of mRNA reverse transcription were predenaturation at 95 °C for 2 min, denaturation at 95 °C for 10 s, annealing at 60 °C for 15 s, extension at 68 °C for 20 s, and 40 cycles of repetition. The cDNA of the miRNA was synthesized using miRNA First Strand cDNA Synthesis (Sangon Biotech). The cDNA gene amplification conditions of miRNA reverse transcription were predenaturation at 95 °C for 10 min, denaturation at 95 °C for 10 s, annealing at 58 °C for 20 s, extension at 72 °C for 10 s, and 40 cycles. The relative expression of miRNA and mRNA was standardized by u6 and β-actin analysed using the 2ΔΔCT method. The primers are shown in Table 2.

**Table 2:** Primer sequence

Gene name	Primer sequence
ALP	F CACGGCGTCCATGAGCAGAAC
	R CAGGCACAGTGGTCAAGGTTGG
COL1A1	F TGTTGGTCCTGCTGGCAAGAATG
	R GTCACCTTGTTCGCCTGTCTCAC
RUNX2	F TCCGCCACCACTCACTACCAC
	R GGAACTGATAGGACGCTGACGAAG
β-actin	F TGGCTCTAACAGTCCGCCTAG
	R AGTGCGACGTGG ACATCCG
OCN	F GGACCCTCTCTCTGCTCACTCTG
	R ACCTTACTGCCCTCCTGCTTGG
PPARγ	F CCATCGAGGACATCCAAGACAACC
	R GTGCTCTGTGACAATCTGCCTGAG
VEGFA	F GCCTTGCCTTGCTGCTCTACC
	R CTTCGTGATGATTCTGCCCTCCTC
FGF2	F GAAGAGCGACCCTCACATCAAGC
	R CCAGGTAACGGTTAGCACACACTC
OPN	F CCAGCCAAGGACCAACTACA
	R GCTGGCAGTGAAGGACTCAT
miR-100-5p	F GGAACCCGTAGATCCGAACTTGTG
	R AACGCTTCACGAATTTGCGT
U6	F CTCGCTTCGGCAGCACA
	R AACGCTTCACGAATTTGCGT

### The extraction of proteins and western blot

Protein lysis buffer (RIPA, Beyotime, China) and protease inhibitor (PMSF, NCM, China) were used to extract protein. The protein concentration of the samples was detected by a BCA protein concentration kit (Beyotime, China). Thirty micrograms protein was loaded into each lane, separated with 10% SDS-PAGE separation gel (Wanlei, China) at 80 V for 30 min and at 120 V for 60 min, transferred to a PVDF membrane (Millipore, USA), and blocked with rapid blocking solution (NCM, China). Then, the protein bands were incubated in primary antibody at 4 °C overnight. The primary antibodies against β-actin, Alix, CD63, CD9, calnexin, PPARγ, Runx2, VEGFA, OPN, and collagen type 1 were purchased from WanleiBio. ALP and TSG101 were purchased from Abcam. FGF2 was purchased from Sabbiotech. OCN and BMPR2 were purchased from Affinity. SMAD1/5/9 and p-SMAD1/5/9 were purchased from Zen-Bio. After the application, the protein bands were washed with TBST three times, and then incubated with secondary antibodies (goat anti-rabbit, 1:8000, Proteintech, China) for 1 h. After dressing, TBST was used for three times, and finally the protein was detected with Zen-Bio ECL (Chengdu, China) reagent.

### Dual luciferase reporter assay

The relationship between the BMPR2 and miR-100-5p was predicted by the bioinformatics database Targetscan 7.2 (www.targetscan.org). The wild-type BMPR2 3′-UTR (WT-BMPR2, containing the miR-100-5p binding site) was inserted into the pMIR-REPORT luciferase vector (Promega Corporation). A cDNA fragment of the mutant sequence of BMPR2-3′-UTR (MT-BMPR2) with a target region was also inserted into the pMIR-REPORT luciferase vector. The sequences of WT-BMPR2 and MT-BMPR2 were further confirmed by sequencing. Then, HEK-293T cells were seeded in a 96-well plate. The cotransfection of WT BMPR2 or MT-BMPR2 with agomiR-100-5p or miR-NC was conducted using Endofectin^TM^-MAX reagent. Relative luciferase activity was further determined with the Dual-Glo Luciferase Assay system.

### Animal study

All experimental and animal care procedures were approved by the Research Ethics Committee of The Affiliated Hospital of Chongqing Medical University and performed in accordance with the guidelines of the National Institutes of Health Guidelines for the Care and Use of Laboratory Animals. A total of 30 female SD rats (8-week-old, 180–200 g) were enrolled in this study and randomly divided into 3 groups: the PBS rats (n = 10), FNF exosome rats (n = 10), and NONFH exosome rats (n = 10) respectively injected with PBS, FNF exosomes, and NONFH exosomes. PBS, FNF exosomes, and NONFH exosomes were injected into rats once every other day for 8 weeks via tail vein. After 8 weeks of injection, all rats were sacrificed to harvest the femoral heads. In every group, 6 femoral heads were grinded with liquid nitrogen and lysed with lysis buffer (RIPA:PMSF = 50:1) to extract bone proteins for western blot analysis. The WB was repeated for 3 times. The S.E.M. bars were calculated using ImageJ and GraphPad software. A microcomputed tomography (micro-CT) (Skyscan1174 X-ray Microtomograph, Bruker, Belgium) was used to scan the rat femoral heads. After scanning, N-Recon software was used for 3-demetional reconstruction of the femoral heads and CT-AN software was used to analyse the osteogenic parameters including BV/TV (bone volume per tissue volume), Tb.Sp (trabecular separation), Tb.Th (trabecular thickness), and Tb.N (trabecular number).

### Histological analyses and immunohistochemistry (IHC)

The collected femoral heads were fixed with 4% paraformaldehyde for 1 week and decalcified with EDTA decalcifying solution. The samples were embedded in paraffin and cut into 5-μm sections, deparaffinized in xylene, rehydrated in a graded series of ethanol solutions, and rinsed in distilled water. H&E staining was performed for histological observation. The expression levels of RUNX2 and CD31 in bone tissue in rat femoral heads were measured using IHC staining.

### Statistical analysis

Statistical analyses were performed using GraphPad PRISM8.0. For all data, normality and homogeneity of variance were detected. All of the measurement data were expressed as mean ± standard error (SEM). For the comparison between two groups, the Student’s t test method was used, while for the comparison among multiple groups, one-way ANOVA and Tukey test method were used. P values ≤ 0.05 was considered statistically significant.

## Results

### Characteristics of NONFH samples, FNF samples, NONFH exosomes, and FNF exosomes

**Fig. 1:**
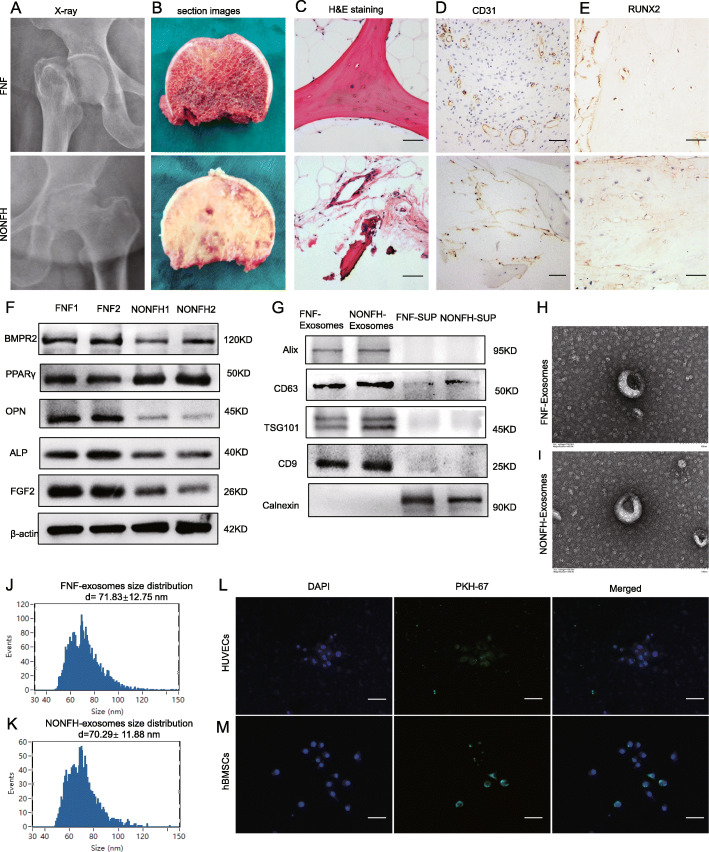
Characteristics of NONFH samples, FNF samples, NONFH exosomes, and FNF exosomes. **A–C** Representative X-rays (**A**), sectional images (**B**), and HE stainings (**C**) of femoral heads from FNF and NONFH patients (scale bar = 200 μm). **D, E** IHC stainings of CD31 (**D**) and RUNX2 (**E**) were conducted in femoral heads of FNF and NONFH patients (scale bar = 50 μm). **F** The expression of PPARγ, OPN, ALP, and FGF2 were measured by western blotting. **G** The expression of CD63, CD9, Alix, calnexin and TSG101 in FNF exosomes, NONFH exosomes, and the FNF-supernatants (FNF-SUP) and NONFH-supernatants (NONFH-SUP) were examined by western blotting. **H, I** TEM images displayed the double membrane and discoid shape of FNF exosomes (**H**) and NONFH exosomes (**I**) (scale bar = 100 nm). **J, K** Particle size distributions of FNF exosomes (**J**) and NONFH exosomes (**K**) were measured by NTA. **L, M** Observation of PKH-67-labeled exosomes were uptaken into hBMSCs and HUVECs under the fluorescence microscopy, where green (PKH-67) indicates exosomes and blue (DAPI) indicates hBMSCs (**M**) and HUVECs (**L**) (scale bar = 50 μm)

The hip joint X-ray of the NONFH group shows necrosis, collapse, and deformation of the femoral head (Fig. 1A). Figure 1B shows sectional images of femoral head specimens from FNF group and NONFH group. H&E stainings showed normal bone trabeculae in the FNF group and collapse, disorder, fracture, and necrosis of trabeculae in the NONFH group (Fig. 1C). IHC showed that the expression of CD31 (Fig. 1D) and RUNX2 (Fig. 1E) were notably decreased in necrotic bone tissue. The western blotting results showed downregulation of osteogenic and angiogenic markers (ALP, OPN, and FGF2) and upregulation of adipogenic marker-PPARγ (Fig. 1F). All of these data conformed to the diagnosis of NONFH by FICAT classification, and IHC slices and WB showed a decrease in osteogenesis and angiogenesis and an augmentation of adipogenesis in necrotic tissues.

To characterize the purified exosome fractions, TEM, NTA, and the exosomal markers analysis were used. Transmission electron microscopy (TEM) indicates typical intact spherical homogeneous morphology of FNF exosomes and NONFH exosomes with diameters about 100 nm (Fig. 1G, H). The results from NTA showed diameter distribution with an average dimension of 71.83 ± 12.75 nm in FNF exosomes and 70.29 ± 11.88 nm in NONFH exosomes (Fig. 1I, J). Figure 1K showed the expression of the exosomal markers CD63, CD9, Alix, calnexin, and TSG-101 in exosomes and supernatant. The uptake assay showed that exosomes could be endocytosed by hBMSCs (Fig. 1L) and HUVECs (Fig. 1M). These data indicate that exosomes were successfully isolated from normal and necrotic bone tissues and can be absorbed by hBMSCs and HUVECs.

### NONFH exosomes inhibited osteogenesis of hBMSCs and angiogenesis of VECs, promoting adipogenesis of hBMSCs

**Fig. 2:**
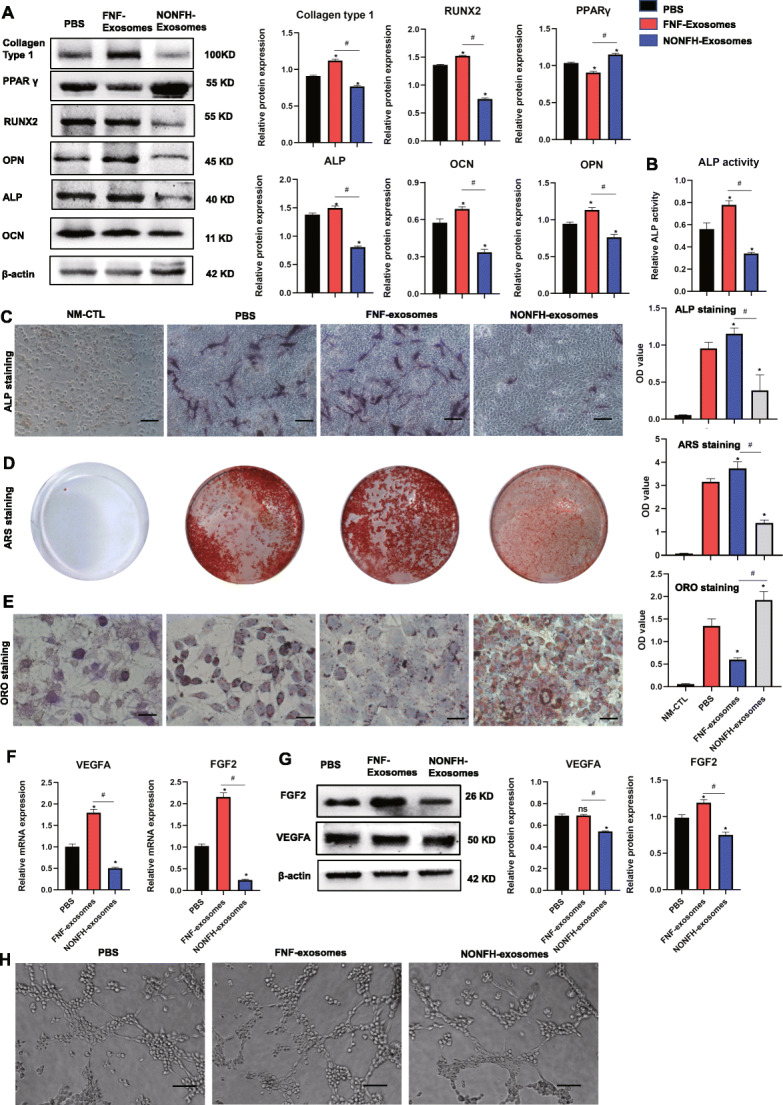
NONFH exosomes inhibited osteogenic differentiation and promoted adipogenic differentiation of hBMSCs. **A** The expression of collagen type 1, OCN, RUNX2, ALP, OPN, and PPARγ were measured by WB in hBMSCs treated with PBS, FNF exosomes, or NONFH exosomes. **B, C** ALP activity and ALP staining of hBMSCs (scale bar = 100 μm). **D** Alizarin Red S staining of hBMSCs. **E** Oil Red O staining of hBMSCs (scale bar = 50 μm). **F, G** The expression of VEGFA and FGF2 were measured by RT-PCR (**F**) and western blotting (**G**). **H** Tube formation assay of HUVECs (scale bar = 200 μm). NM-CTL, controls for differentiation. *P < 0.05, versus PBS group; #P < 0.05, versus FNF exosome group. All data were expressed as mean ± SEM

To investigate the effect of FNF exosomes and NONFH exosomes on the osteogenesis and adipogenesis of hBMSCs, we examined osteogenic and adipogenic markers, including OCN, OPN, ALP, RUNX2, collagen type 1, and PPARγ, in the hBMSCs treated with PBS, FNF exosomes, or NONFH exosomes for 7 days. The results of western blotting and RT-PCR showed that the expression of osteogenic markers was decreased in NONFH exosome group, while the expression of PPARγ was increased (Fig. 2A, B). In addition, the ALP activity assay and ALP staining demonstrated the suppressing effect of NONFH exosomes on ALP activity of hBMSCs (Fig. 2C, D). Alizarin Red S staining also showed the significant decrease of calcium deposition on the surface of the hBMSCs in the NONFH exosome group (Fig. 2E). The Oil Red O staining showed that the NONFH exosomes promoted the formation of lipid droplets in hBMSCs (Fig. 2F). These data indicate that NONFH exosomes could inhibit the osteogenic differentiation of hBMSCs, accompanied by an augmentation in the adipocyte differentiation of hBMSCs.

Next, we measured the angiogenesis of the HUVECs treated with the NONFH exosomes by employing RT-PCR, WB, and a tube formation assay. Figure 2G and H show the downregulation of VEGFA and FGF2 in HUVECs treated with NONFH exosomes. The tube formation assay showed that the tube formation of HUVECs was significantly decreased in NONFH exosome group (Fig. 2I). These data indicated that the NONFH exosomes influenced the angiogenesis of HUVECs.

### NONFH exosomes inhibited the migration ability of hBMSCs and HUVECs

**Fig. 3:**
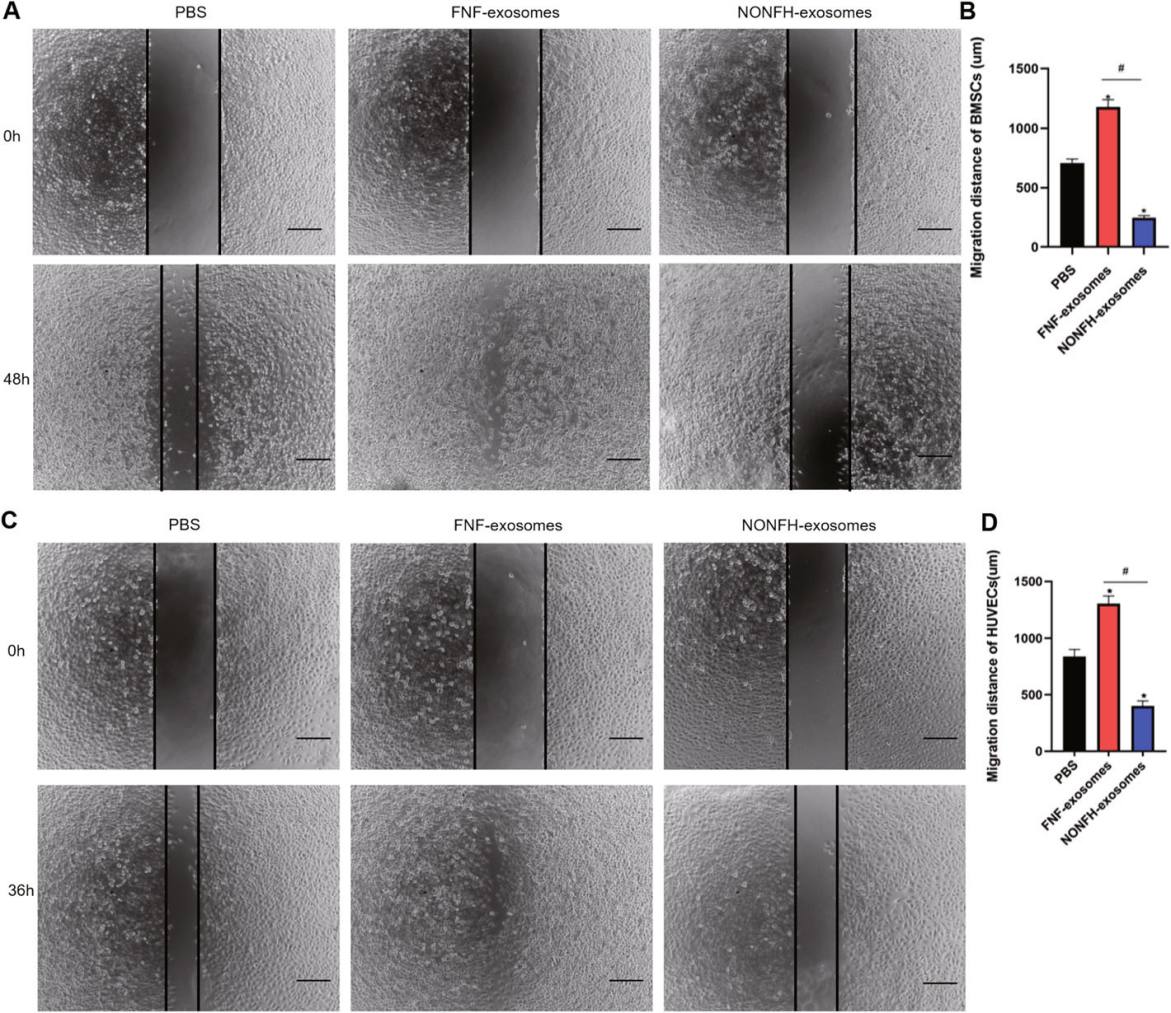
NONFH exosomes inhibited the migration ability of hBMSCs and HUVECs. **A, B** Wound healing assay of hBMSCs (**A**) and its quantitative analysis (**B**). **C, D** Wound healing assay of HUVECs (**C**) and its quantitative analysis (**D**). Scale bar = 200 μm. *P < 0.05, versus PBS group; #P < 0.05, versus FNF exosome group. All data were expressed as mean ± SEM

Next, we conducted wound healing assays to investigate the effects of the NONFH exosomes on the migration of hBMSCs and HUVECs. The pictures photographed at 0 h and the terminal point 48 h of hBMSCs showed that the migration of hBMSCs was suppressed by the NONFH exosomes and promoted by the FNH-exosomes (Fig. 3A, B). In addition, the images taken at 0 h and 36 h indicated that the migration of HUVECs was also inhibited in the NONFH exosome group (Fig. 3C, D). These results showed that the migratory distances of hBMSCs and HUVECs in the NONFH exosome group were decreased.

### NONFH exosomes could lead to NONFH-like damage on rats

**Fig. 4:**
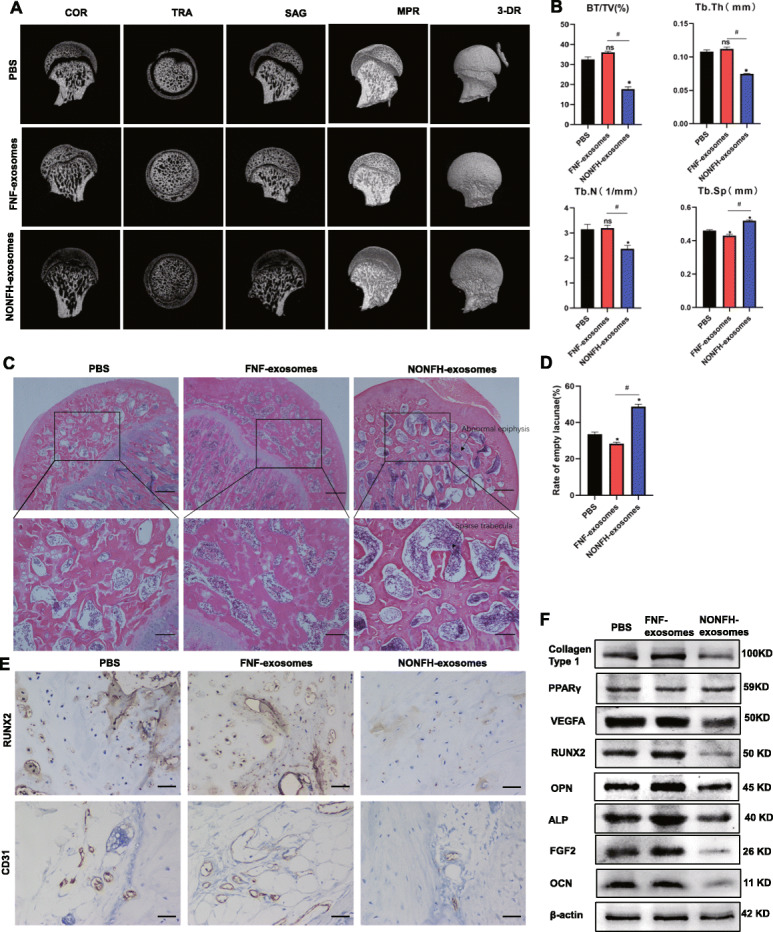
NONFH exosomes could lead to NONFH-like damage on rats. **A** COR, TRA, SAG, MPR, and 3-DR images of rat femoral heads. COR, coronal; TRA, transverse; SAG, sagittal; MPR, multiplanar reconstruction; 3-DR, three-dimensional reconstruction. **B** Quantitative analysis of micro-CT scanning. BV/TV, bone volume per tissue volume; Tb.N, trabecular number; Tb.Sp, trabecular separation; Tb.Th, Trabecular thickness. **C** H&E staining of the femoral heads (scale bar = 200 μm). **D** Quantitative analysis of marrow cavity. **E** The expression of RUNX2 and CD31 in the femoral head of rats were measured by IHC staining (scale bar = 50 μm). **F** Western blot was used to detect the expression of collagen type 1, VEGFA, FGF2, RUNX2, OPN, PPARγ, ALP, and OCN in femoral heads of rats. *P < 0.05, versus PBS group; #P < 0.05, versus FNF exosome group. All data were expressed as mean ± SEM

Furthermore, the NONFH exosomes, the FNF exosomes, or PBS was injected into rats via tail vein to explore the effects of the NONFH exosomes in vivo. The micro-CT scanning results suggested that about 60% rats in NONFH exosome group had bone tissue changes, including subchondral bone lesion, collapse, and malformed shape of the femoral head (Fig. 4A). Qualitative analyses of all the micro-CT parameters showed that BV/TV, Tb.Th, and Tb.N were decreased with the augmentation of Tb.Sp in the rats of NONFH exosome group (Fig. 4B). H&E staining revealed the NONFH-like damage in the NONFH exosome group, including subchondral bone lesions, more marrow cavities, and sparser trabeculae (Fig. 4C, D). Next, IHC staining showed the reduction of RUNX2 and VEGFA levels (Fig. 4E). The results of western blotting showed that the expression of collagen type 1, VEGFA, FGF2, RUNX2, OPN, ALP, and OCN were downregulated in the rats of the NONFH exosome group, while the expression of PPARγ was upregulated (Fig. 4F, G). These data indicate that NONFH exosomes induced the NONFH-like damage with the reduction of osteogenesis and angiogenesis and augmenting of adipogenesis in vivo.

### MiR-100-5p was upregulated in NONFH exosomes

**Fig. 5:**
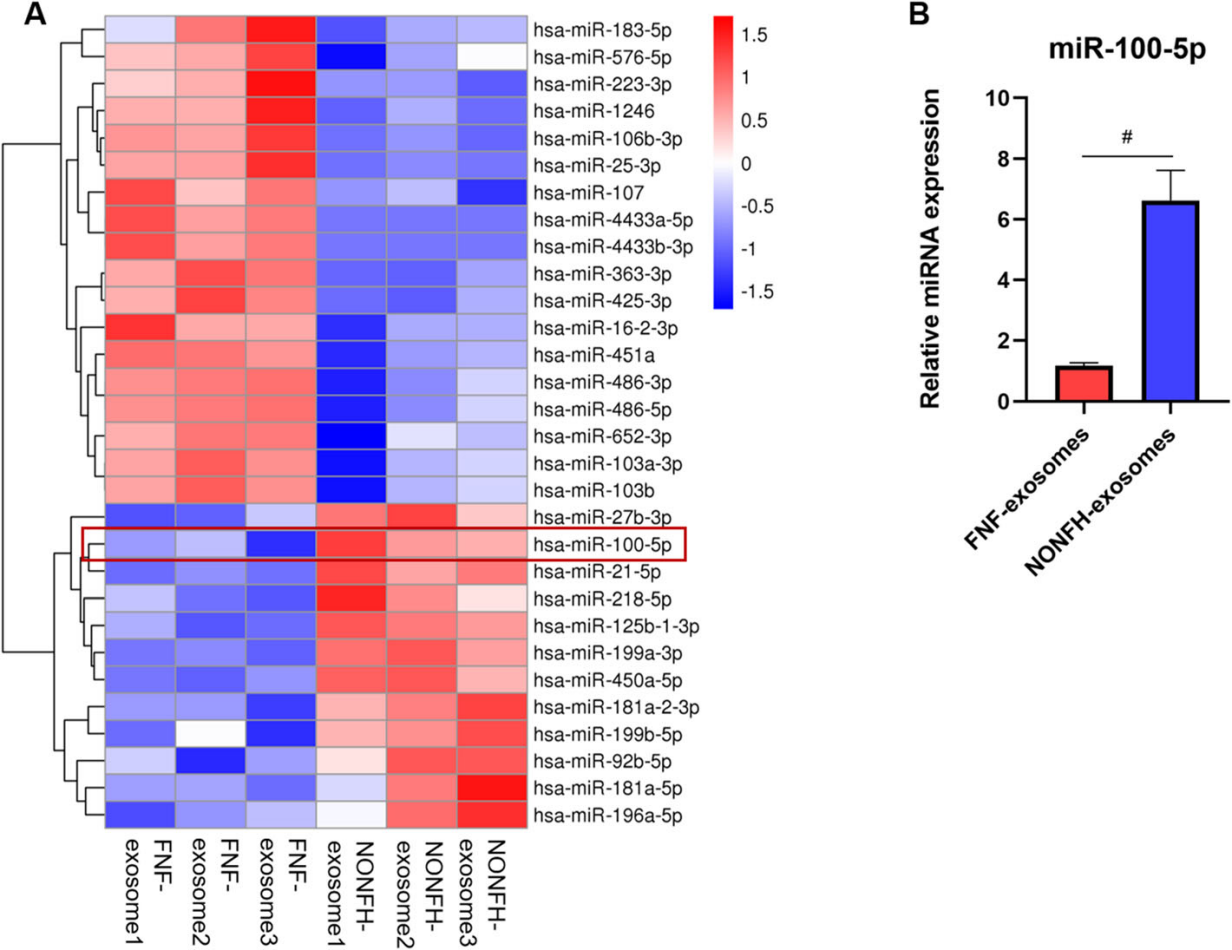
MiR-100-5p was upregulated in NONFH exosomes. **A** Heatmap of the miRNA sequencing. **B** RT-PCR was used to detect the expression of miR-100-5p in the FNF exosomes and the NONFH exosomes. #P < 0.05, versus FNF exosome group. All data were expressed as mean ± SEM

To investigate whether the expression of miRNAs was different between the NONFH exosomes and the FNF exosomes, we performed microRNA sequencing. We analysed differentially abundant miRNAs following the criteria of *P* value < 0.05. Differentially abundant miRNAs between the NONFH exosomes and the FNF exosomes were visualized using volcano plot (Fig. 5A) and heatmap (Fig. 5B). A total of 30 differentially expressed miRNAs were identified, including 12 miRNAs with upregulated expression and 18 miRNAs with downregulated expression. The results of GO enrichment and KEGG pathway enrichment analysis were separately displayed in Fig. 5C, D). RT-PCR was further used to measure the expression of miR-100-5p, and the result was similar to the results of microRNA sequencing (Fig. 5E).

### MiR-100-5p inhibited osteogenesis of hBMSCs and angiogenesis of HUVECs, promoting adipogenesis of hBMSCs

**Fig. 6:**
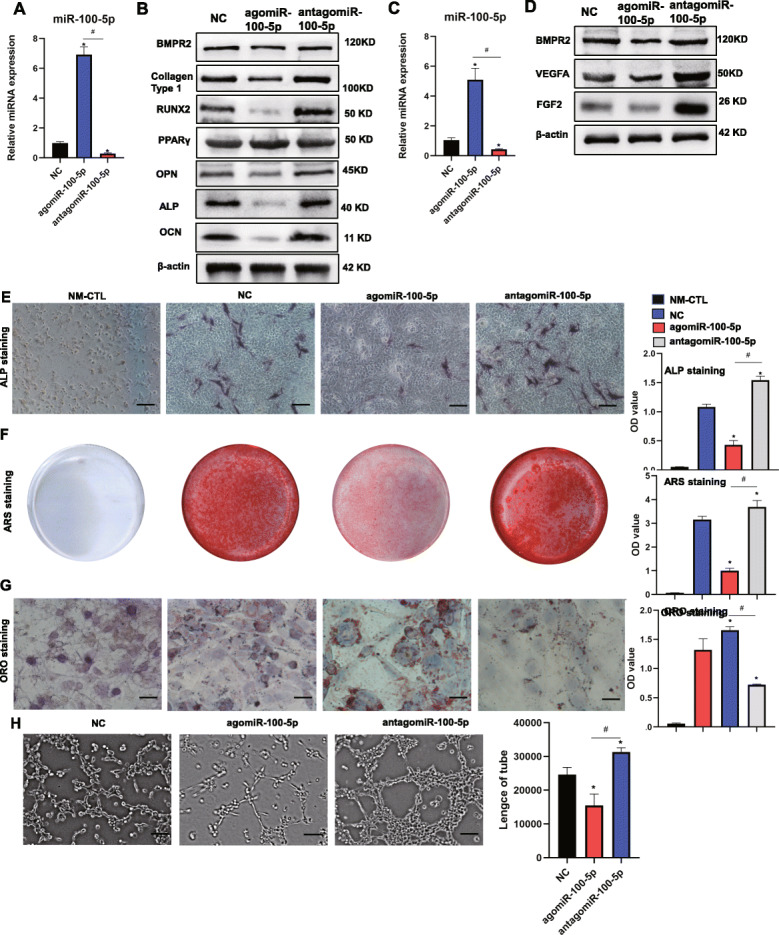
MiR-100-5p inhibited osteogenesis of hBMSCs and angiogenesis of HUVECs, promoting adipogenesis of hBMSCs. **A** RT-PCR was used to detect the expression of miR-100-5p in hBMSCs. **B** WB was used to detect the expression of BMPR2, collagen type 1, OCN, RUNX2, ALP, OPN, and PPARγ in hBMSCs. **C** The expression of miR-100-5p in HUVECs. **D** WB was used to measure the expression of BMPR2, FGF2, and VEGFA in HUVECs. **E** ALP staining of hBMSCs (scale bar = 100 μm). **F** Alizarin Red S staining of hBMSCs after cultured in ODM for 14 days. **G** Oil Red O staining of hBMSCs (scale bar = 50 μm). **H** Tube formation assay of HUVECs (scale bar = 200 μm). NM-CTL, controls for differentiation. *P < 0.05, versus NC group; #P < 0.05, versus agomiR-100-5p group. All data were expressed as mean ± SEM

As the results of miRNA sequencing and RT-qPCR showed that miR-100-5p expression was upregulated in the NONFH exosomes, we studied the effects of miR-100-5p on hBMSCs and HUVECs. We found that the transfection of agomiR-100-5p and antagomiR-100-5p could separately increase and decrease the expression of miR-100-5p in hBMSCs and HUVECs, respectively (Fig. 6A, C). The results of western blotting showed that the expression of OCN, RUNX2, ALP, and collagen type 1 were significantly reduced in hBMSCs transfected with agomiR-100-5p with the augmentation of PPARγ (Fig. 6B). ALP staining showed that the ALP activity of hBMSCs was inhibited in the agomiR-100-5p group (Fig. 6E). ARS staining showed that the mineralization of hBMSCs was reduced in the agomiR-100-5p group (Fig. 6F). The results of Oil Red O staining showed the formation of lipid droplets was promoted in the agomiR-100-5p group (Fig. 6G). The results demonstrated that the expression of VEGFA and FGF2 were significantly decreased in HUVECs transfected with agomiR-100-5p (Fig. 6H). Next, we conducted tube formation assays, and the results showed that agomiR-100-5p inhibited tube formation of HUVECs (Fig. 6H).

### MiR-100-5p inhibits osteogenesis of hBMSCs and angiogenesis of HUVECs by targeting BMPR2 and inhibiting BMPR2/smad1/5/9 pathway

**Fig. 7:**
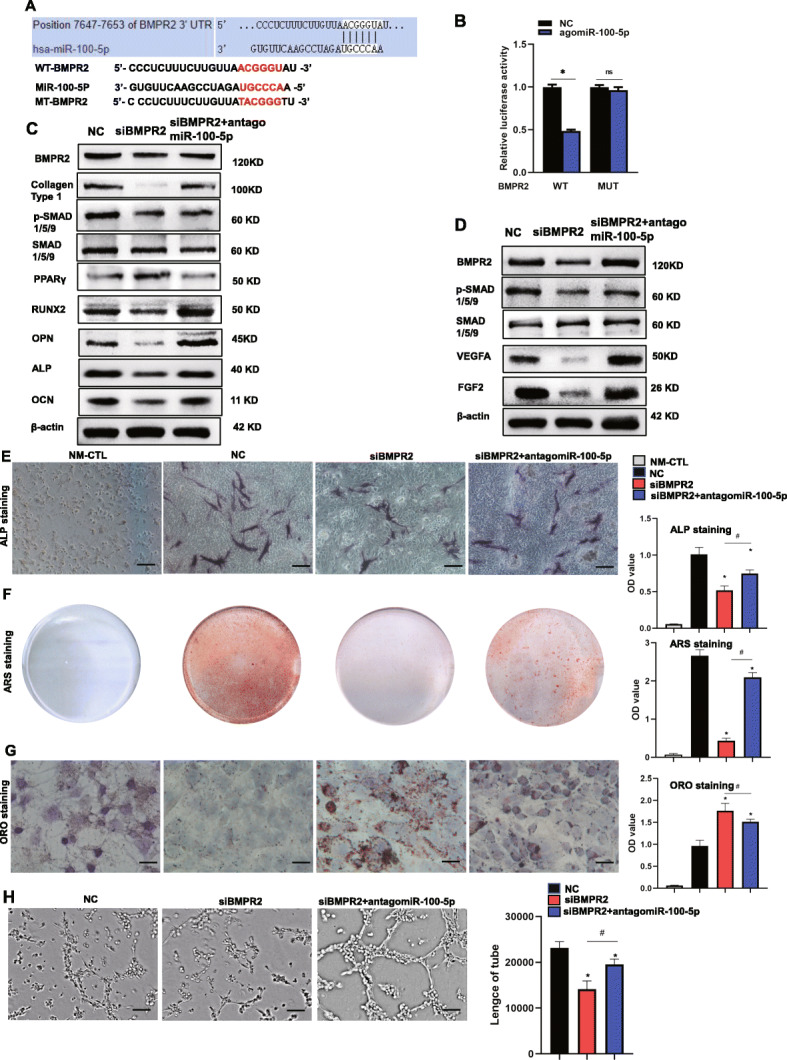
MiR-100-5p inhibits osteogenesis of hBMSCs and angiogenesis of HUVECs by targeting BMPR2 and inhibiting the BMPR2/smad1/5/9 pathway. **A** The target gene of miR-100-5p was predicted using Targetscan 7.2. **B** The targeting relationship between miR-100-5p and BMPR2 was verified by a luciferase assay. **C** Western blotting was used to measure the expression of collagen type 1, BMPR2, SMAD1/5/9, p-SMAD1/5/9, OCN, RUNX2, ALP, OPN, and PPARγ. **D** Western blotting was used to measure the expression of BMPR2, SMAD1/5/9, p-SMAD1/5/9, FGF2, and VEGFA in HUVECs. **E** ALP staining of hBMSCs (scale bar = 100 μm). **F** Alizarin Red S staining of hBMSCs. **G** Oil Red O staining of hBMSCs (scale bar = 50 μm). **H** Tube formation assay of HUVECs (scale bar = 200 μm). NC, transfection with NC; siBMPR2, transfection with small interfering RNA of BMPR2; siBMPR2 + antagomiR-100-5p, transfection with small interfering RNA of BMPR2 and antagonist of miR-100-5p; NM-CTL, controls for differentiation. *P < 0.05, versus NC group; #P < 0.05, versus agomiR-100-5p group. NS P>0.05, versus NC group. All data were expressed as mean ± SEM

We predicted the target genes using TargetScan 7.2. We found that there was a binding side between miR-100-5p and BMPR2 (Fig. 7A). This finding was further confirmed by a dual luciferase reporter assay (Fig. 7B) and western blotting (Fig. 6B, D), the results of which showed that overexpression of miR-100-5p significantly suppressed the luciferase activity of 3′-UTR in the wild-type compared with the miR-NC group, whereas no differences in luciferase activity of 3′-UTR were observed in the mutant-type. The above results confirmed that BMPR2 was the target gene of miR-100-5p.

To further investigate the function of BMPR2, we silenced the expression of BMPR2 to evaluate changes in differentiation of hBMSCs and HUVECs. The silencing of BMPR2 significantly reduced osteogenic differentiation, as evidenced by decreased expression of osteogenic markers (Fig. 7C), diminished ALP activity (Fig. 7E), and decreased mineralization capacity (Fig. 7F) with the augmentation of adipogenic marker and lipid droplets in hBMSCs (Fig. 7C, G). The downregulation of miR-100-5p (antagomiR-100-5p) partly reversed the negative effect of siBMPR2 on osteogenesis and the positive effect on adipogenesis of hBMSCs. The silencing of BMPR2 in HUVECs significantly reduced the expression of FGF2 and VEGFA and the number of formed tubes (Fig. 7D, H). From the KEGG database, we found that BMPR2 was associated with the BMP-SMAD pathway. According to the previous study, BMP-SMAD signalling was significantly associated with osteogenesis and angiogenesis [37, 38]. We next detected BMPR2, SMAD1/5/9, and phosphorylated SMAD1/5/9 (p-SMAD1/5/9) in hBMSCs and HUVECs (Fig. 7C, D). It was obvious that siBMPR2 influenced the expression of these proteins in hBMSCs and HUVECs. Silencing of BMPR2 suppressed the osteogenesis of hBMSCs and angiogenesis of HUVECs via inactivating the BMPR2/SMAD1/5/9 pathway. The suppression of miR-100-5p expression could partly rescue the suppression of osteogenesis of hBMSCs and angiogenesis of HUVECs caused by siBMPR2. Taken together, these data suggest that the BMPR2/SMAD1/5/9 pathway is involved in osteogenesis and angiogenesis through interaction with miR-100-5p.

### AntagomiR-100-5p rescued the suppression of osteogenesis of hBMSCs and angiogenesis of HUVECs caused by NONFH exosomes

**Fig. 8:**
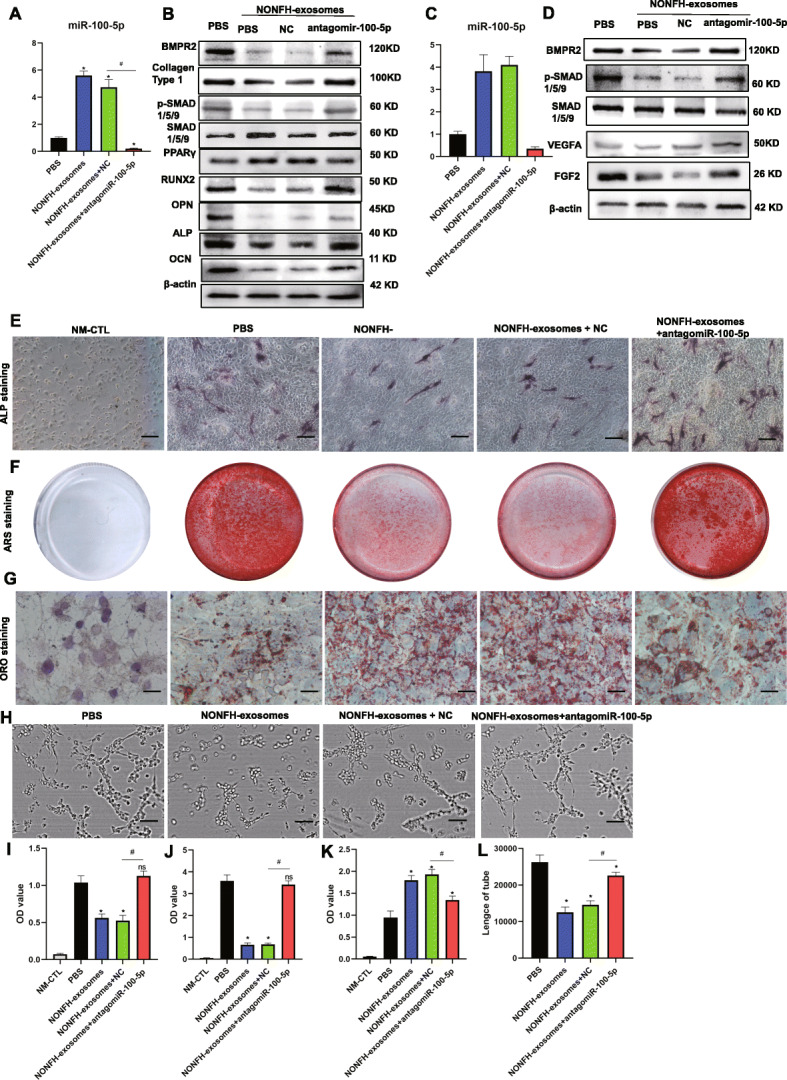
AntagomiR-100-5p rescued the suppression of osteogenesis of hBMSCs and angiogenesis of HUVECs caused by NONFH exosomes. **A** The expression of miR-100-5p in hBMSCs was measured by RT-PCR. **B** WB was used to detect the expressions of BMPR2, SMAD1/5/9, p-SMAD1/5/9, collagen type 1, OCN, RUNX2, ALP, OPN, and PPARγhBMSCs. **C** The expression of miR-100-5p in HUVECs was measured by RT-PCR. **D** WB was used to detect the expression of BMPR2, BMPR2, SMAD1/5/9, p-SMAD1/5/9, FGF2, and VEGFA in HUVECs. **E** ALP staining of hBMSCs (scale bar = 100 μm). **F** Alizarin Red S staining of hBMSCs after cultured in ODM for 14 days. **G** Oil Red staining of hBMSCs (scale bar = 50 μm). **H** Tube formation assay of HUVECs (scale bar = 200 μm). **I** Semiquantitative analysis of ALP staining. **J** Quantitative analysis of ARS staining. **K** Quantitative analysis of Oil Red O staining. **L** Quantitative analysis of tube formation. PBS, treatment with PBS; NONFH exosomes, treatment with NONFH exosomes; NONFH exosomes+ NC, treatment with NONFH exosomes and NC; treatment with NONFH exosomes +antagomiR-100-5p, treatment with NONFH exosomes and antagonist of miR-100-5p; NM-CTL, controls for differentiation. *P < 0.05, versus NC group; #P < 0.05, versus agomiR-100-5p group. NS P>0.05, versus PBS group All data were expressed as mean ± SEM

The data of Fig. 7 show that antagomiR-100-5p remedied the decreased osteogenesis of hBMSCs and angiogenesis of HUVECs caused by siBMPR2. Then we further investigated the effect of antagomiR-100-5p on the NONFH exosome-induced NONFH-like impairment model in hBMSCs and HUVECs. The results of RT-PCR showed that the expression of miR-100-5p in hBMSCs and HUVECs were downregulated by antagomiR-100-5p (Fig. 8A, C). To assess the effects of antagomiR-100-5p on the osteogenesis of hBMSCs treated with NONFH exosomes, the osteogenic markers were measured by western blotting (Fig. 8B). The NONFH-induced expression of collagen type 1, OCN, ALP, OPN, Runx2, BMPR2, and p-SMAD1/5/9 was dramatically decreased and could be partly rescued by the treatment of antagomiR-100-5p. The results suggested that antagomiR-100-5p could partly restore the impaired osteogenesis in hBMSCs caused by NONFH exosomes. In addition, ALP staining and Alizarin Red S staining demonstrated a similar effect of antagomiR-100-5p on ALP activity and mineralization (Fig. 8E, F). However, the expression of PPARγ and the formation of lipid droplets were increased in hBMSCs after treatment with NONFH exosomes, while antagomiR-100-5p could partly suppress the upregulation of PPARγ and formatted lipid droplets of hBMSCs caused by NONFH exosomes (Fig. 8B, G). In addition, western blotting showed that transfection with antagomiR-100-5p could partly rescue the downregulation of FGF2, VEGFA, BMPR2, and p-SMAD1/5/9 in HUVECs caused by NONFH exosomes (Fig. 8D). The tube formation assay showed the same trend (Fig. 8H). These data demonstrated that antagomiR-100-5p were able to restore the influence of the NONFH exosomes on the differentiation of hBMSCs and HUVECs.

## Discussion

The reduced angiogenesis of VECs and osteogenesis of BMSCs play an integral role in the initiation and progression of NONFH. The early diagnosis and treatment of this disease is difficult. Thus, it is important to elucidate the details of the molecular mechanisms to identify novel therapeutic strategies. In the present study, we observed that the NONFH exosomes could inhibit osteogenesis and angiogenesis in vitro and in vivo and that miR-100-5p expression was upregulated in the NONFH exosomes. Moreover, we revealed the key role of the NONFH exosomes and exosomal miR-100-5p in mediating osteogenesis and angiogenesis and provided fruitful useful underlying targets to prevent the development of NONFH. In our study, we confirmed their harmful roles in NONFH and identified new target molecules and the mechanisms by which exosomal miR-100-5p suppressed osteogenesis and angiogenesis.

Exosomes, a member of extracellular vesicles, serve as crucial vehicles for intercellular communication and have been documented in many bone and joint diseases. The osteogenic and angiogenic effects of exosomes on NONFH have also been demonstrated in previous studies. Fang et al. found that BMSC-derived exosomes can prevent steroid-induced osteonecrosis of the femoral head by enhancing the osteogenic differentiation of BMSCs from rats with NONFH [20]. Zuo et. al. transfected lentiviral of miR-26a into CD34+ stem cells, collected miR-26a overexpressing exosomes from CD34+ stem cells (miR-26a-CD34+ exosomes) and found that the miR-26a-CD34+ exosomes could prevent glucocorticoid-induced osteonecrosis of the femoral head by promoting angiogenesis and osteogenesis in vitro and in vivo [39]. Li et al. found that extracellular vesicles derived from BMSCs significantly promoted the osteogenesis of BMSCs and angiogenesis of HUVECs [6]. However, no study has reported the effect and function of exosomes derived from necrotic bone tissues of patients with NONFH. In addition, elucidating and targeting the key molecules that mediate the reduced osteogenesis and angiogenesis in necrotic bone tissues might be a promising way to prevent NONFH. In our study, we first extracted the NONFH exosomes and studied their effects. Excitingly, we found that the NONFH exosomes could inhibit osteogenic differentiation and promote adipogenic differentiation of hBMSCs as well as suppress angiogenic differentiation of HUVECs. We also found that the injection of the NONFH exosomes resulted in a NONFH-like damage in rats. Thus, our findings not only uncovered a novel mechanism mediating NONFH, but also highlighted the clinical significance of NONFH exosomes in NONFH treatment.

To investigate the mechanism of action of NONFH exosomes’ impacts on osteogenesis and angiogenesis, we conducted a miRNA sequence. Many differentially expressed miRNAs were identified in exosomes from multiple MSCs and the broad biological significance importance of exosomal miRNAs on osteogenesis and angiogenesis of NONFH has been investigated, including miR-21, miR-26a, miR-148a-3p, miR-451-5p, and miR-365a-5p [18, 21, 39–41]. The downstream target genes of these miRNAs include PTEN, PDCD4 PAI-1, and SAV1. However, no study has thoroughly explored the expression of miR-100-5p in the NONFH exosomes or the role of exosomal miR-100-5p in the pathogenesis of NONFH. In our study, we found that the level of miR-100-5p expression in the NONFH exosomes was significantly higher than that in the FNF exosomes, a finding that was validated by qPCR. Previously, miR-100-5p was reported to be closely connected with angiogenesis and osteogenesis [42, 43]. In our study, we first found that the overexpression of miR-100-5p could strongly inhibit osteogenesis of hBMSCs and angiogenesis of HUVECs as well as to promote adipogenesis of hBMSCs. Moreover, it was observed that the silencing of miR-100-5p could significantly reverse the suppression of osteogenesis and angiogenesis caused by the NONFH exosomes. Thus, our findings provide new insight into the regulation of osteogenesis, adipogenesis, angiogenesis, and progression of NONFH.

To study the molecular mechanism by which miR-100-5p regulates the differentiation of hBMSCs and HUVECs, we performed a search with TargetScan, which revealed that BMPR2 might be a possible target with 9 nt nonconsecutive match site complementary to miR-100-5p in its 3″UTR. To confirm this prediction, we conducted a dual luciferase reporter assay and identified BMPR2 as a direct target of miR-100-5p. Moreover, WB showed that upregulation of miR-100-5p expression led to downregulation of BMPR2 at the protein level, whereas functional inhibition of miR-100-5p led to derepression of BMPR2, strongly suggesting that BMPR2 is regulated by miR-100 during osteogenic differentiation. Our data clarufied that the silencing of BMPR2 expression inhibited osteogenesis and angiogenesis, while these effects were rescued by antagomiR-100-5p. BMPR2, also named bone morphogenetic protein receptor 2, is an important receptor of the BMP family that can promote osteoblastic differentiation and angiogenesis as well as inhibiting the adipogenesis. BMPR2 can directly phosphorylate and activate BMPR1; then, active BMPR1 phosphorylates SMAD1/5/9 and promotes the binding of SMAD1/5/9 and SMAD4 and nuclear translocation, thus promoting the activation of osteogenesis and angiogenesis and inhibiting adipogenesis. Yeh et al. detected the RNA expression of BMPR2 in the blood of 220 patients treated with glucocorticoids for SLE (55 with NONFH and 165 without NONFH) and found that BMPR2 was reduced by more than 50% in the blood of the patients with NONFH [44]. In the present study, we found that the expression of BMPR2 significantly decreased in the necrotic region of the patients with NONFH and the hBMSCs, HUVECs, and rats treated with the NONFH exosomes. Additionally, phosphorylation of SMAD1/5/9 was reduced in necrotic zones and the hBMSCs and HUVECs treated with the NONFH exosomes as well as with overexpression of miR-100-5p, which indicated the central role of the exosomal miR-100-5p-BMPR2 axis.

## Conclusion

In conclusion, our study demonstrated that NONFH exosomes could lead to NONFH-like damage in vitro and in vivo. In addition, we found that the upregulation of NONFH exosomal miR-100-5p suppressed the differentiation of hBMSCs and HUVECs by targeting BMPR2 and inactivating the BMPR2/Smad1/5/9 signalling pathway. Furthermore, our study would also provide clues for us to further explore the pathogenesis and therapeutic strategies for NONFH and the failure of cell therapy.

## Supplementary Information

**Additional file 1: S1.** RT-PCR was used to detect the expressions of collagen type 1, OCN, RUNX2, ALP, OPN and PPARγ in hBMSCs treated with PBS, FNF exosomes, or NONFH exosomes.

**Additional file 2:.** S2.Semiquantitative analysis of western blot in Figure 4F.

**Additional file 3: S3.** Supplementary material of Figure 5A Volcano map of the miRNA sequencing. B GO enrichment. C KEGG pathway enrichment. D scatter diagram of Figure 5B.

**Additional file 4: Supplementary material 4.** The repeated results of western blot.

## Data Availability

All data used and analysed during the current study are available from the corresponding author on reasonable request. All data generated or analysed during this study are included in this article.

## Abbreviations

qRT-PCR: Quantitative real-time polymerase chain reaction
ALP: Alkaline phosphatase
ARS: Alizarin Red S
BCA: Bicinchoninic acid
BMI: Body mass index
BMPR2: Bone morphogenetic protein receptor 2
BMSCs: Bone marrow mesenchymal stem cells
BSA: Bovine serum albumin
BV/TV: Bone volume per tissue volume
DLS: Dynamic light scattering
FBS: Fetal bovine serum
FGF2: Fibroblast growth factor 2
FNF: Femoral neck fracture
HE: Hematoxylin and eosin
IHC: Immunohistochemistry
HUVECs: Human umbilical vein endothelial cells
IHC: Immunohistochemistry
KEGG: Kyoto Encyclopedia of Genes and Genomes
NC: Negative control
NONFH: Nontraumatic osteonecrosis of the femoral head
NTA: Nanoparticle tracking analysis
OCN: Osteocalcin
OPN: Osteopontin
PBS: Phosphate buffer saline
PPARγ: Peroxisome proliferator-activated receptor γ
RUNX2: Runt-related transcription factor 2
SDS-PAGE: Sodium dodecyl sulphate polyacrylamide gel electrophoresis
SEM: Standard error of mean
Tb.N: Trabecular number
Tb.Sp: Trabecular separation
Tb.Th: Trabecular thickness
TBST: Tris-buffered saline/Tween-20 buffer
TEM: Transmission electron microscopy
VECs: Vascular endothelial cells
VEGFA: Vascular endothelial growth factor
3′UTR: 3′-Untranslated region
