# Supplementary figures and images for: Exosomal miR-100-5p inhibits osteogenesis of hBMSCs and angiogenesis of HUVECs by suppressing the BMPR2/Smad1/5/9 signalling pathway
Source: Stem Cell Res Ther. 2021 Jul 13;12:390. doi: 10.1186/s13287-021-02438-y (PMC8278698; doi:10.1186/s13287-021-02438-y)

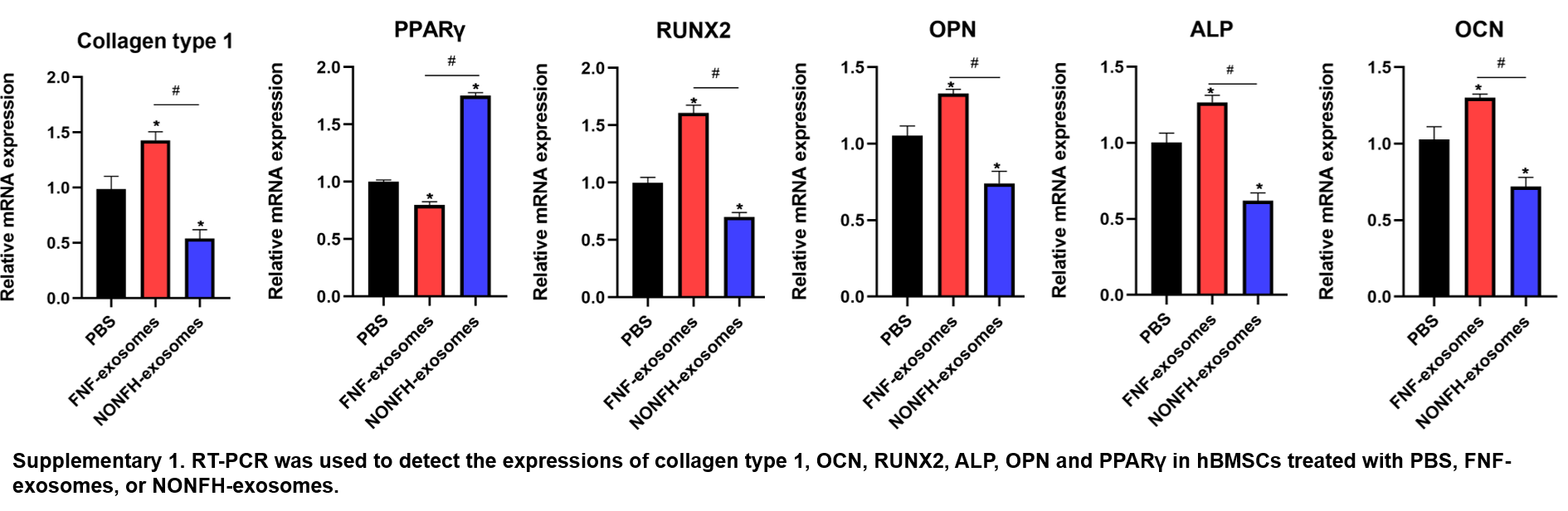

Supplement: Supplementary file 1 — Additional file 1: S1. RT-PCR was used to detect the expressions of collagen type 1, OCN, RUNX2, ALP, OPN and PPARγ in hBMSCs treated with PBS, FNF exosomes, or NONFH exosomes. [file 13287_2021_2438_MOESM1_ESM.tif]

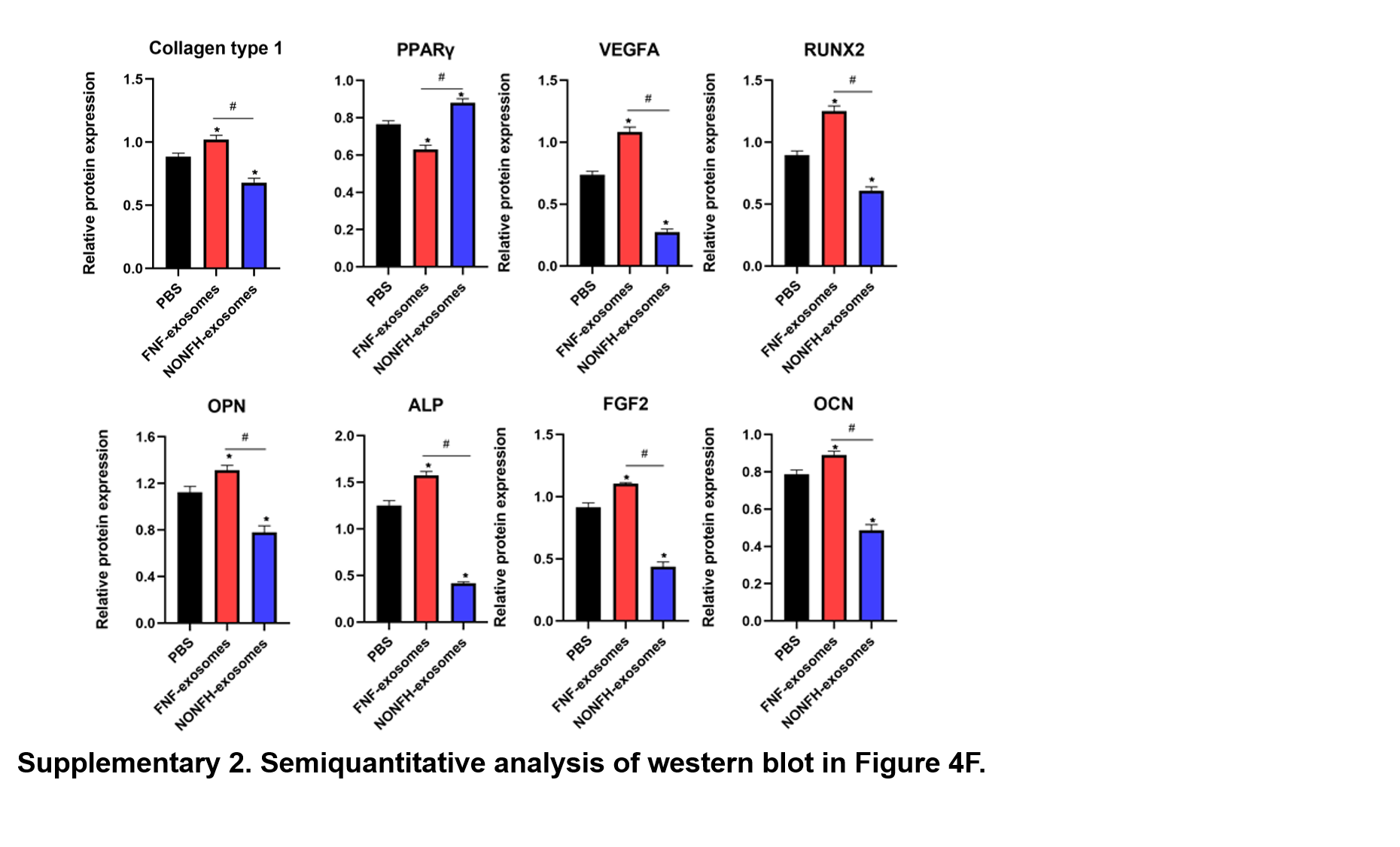

Supplement: Supplementary file 2 — Additional file 2:. S2.Semiquantitative analysis of western blot in Figure 4F. [file 13287_2021_2438_MOESM2_ESM.tif]

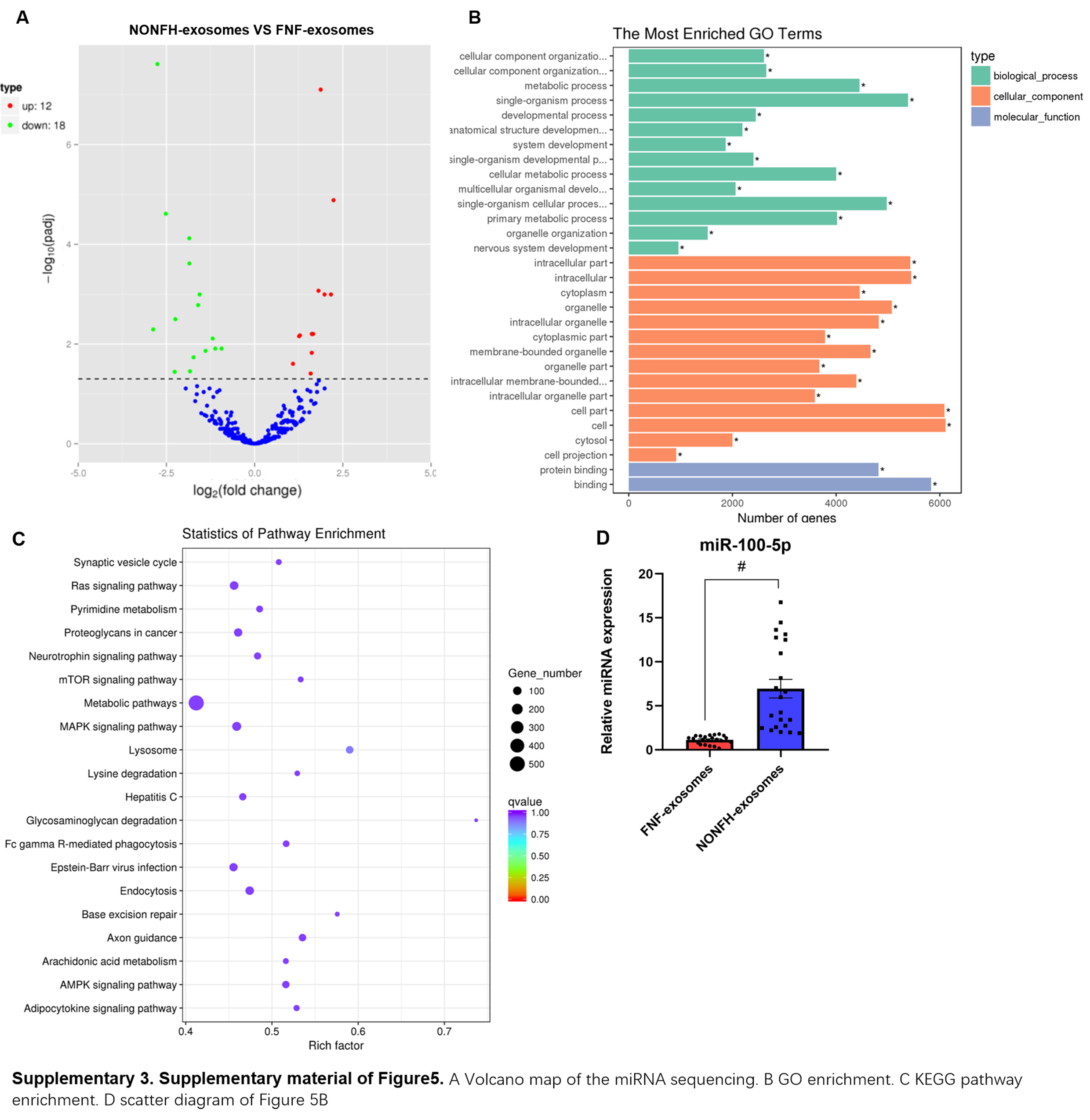

Supplement: Supplementary file 3 — Additional file 3: S3. Supplementary material of Figure 5A Volcano map of the miRNA sequencing. B GO enrichment. C KEGG pathway enrichment. D scatter diagram of Figure 5B. [file 13287_2021_2438_MOESM3_ESM.tif]
