# Supplementary material for: Exosomal miR-100-5p inhibits osteogenesis of hBMSCs and angiogenesis of HUVECs by suppressing the BMPR2/Smad1/5/9 signalling pathway
Source: Stem Cell Res Ther. 2021 Jul 13;12:390. doi: 10.1186/s13287-021-02438-y (PMC8278698; doi:10.1186/s13287-021-02438-y)
Supplement: Supplementary file 4 — Additional file 4: Supplementary material 4. The repeated results of western blot. [file 13287_2021_2438_MOESM4_ESM.pdf]

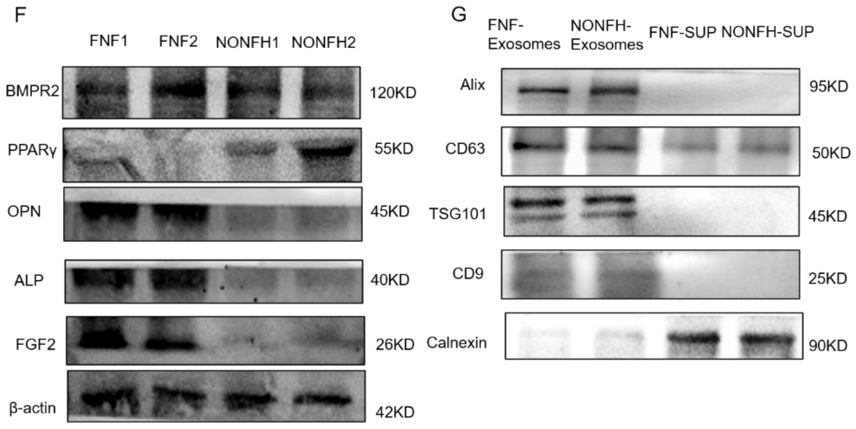

Figure1 F and G repeat 1

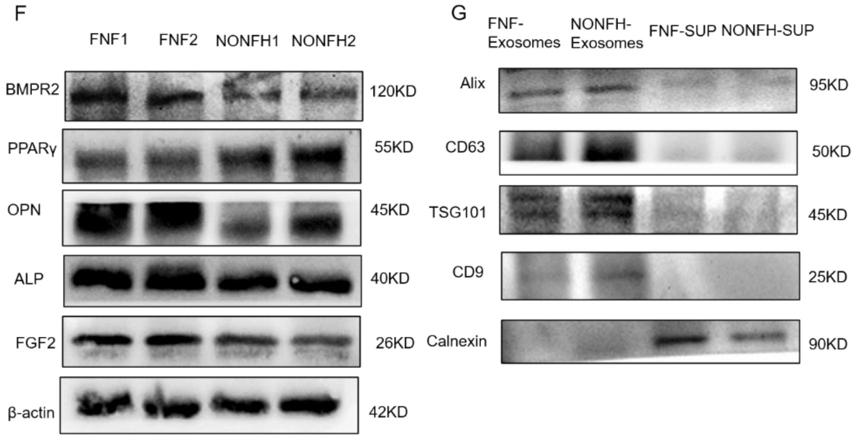

Figure1 F and G repeat 2

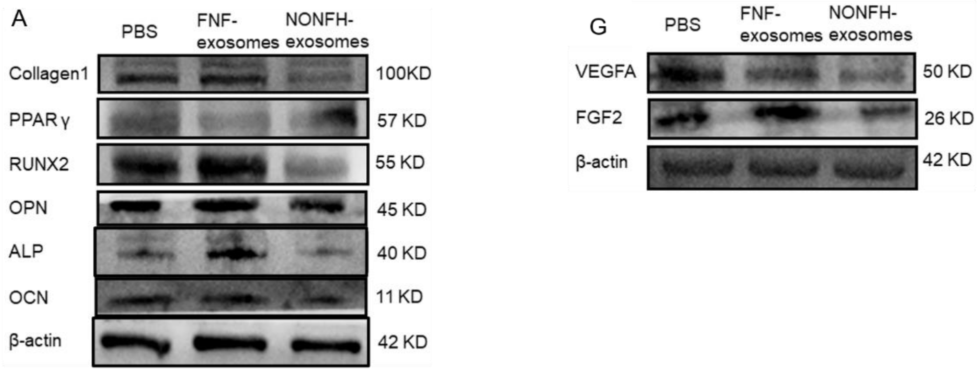

Figure2 A and G repeat 1

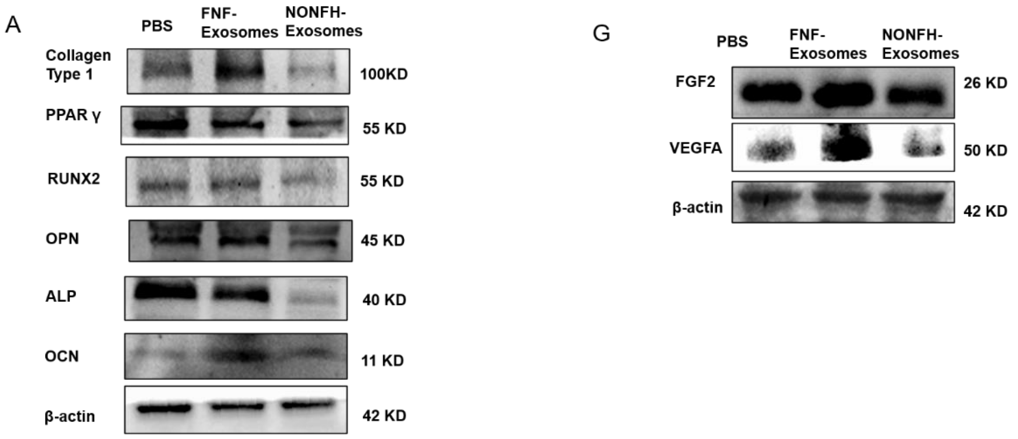

Figure2 A and G repeat 2

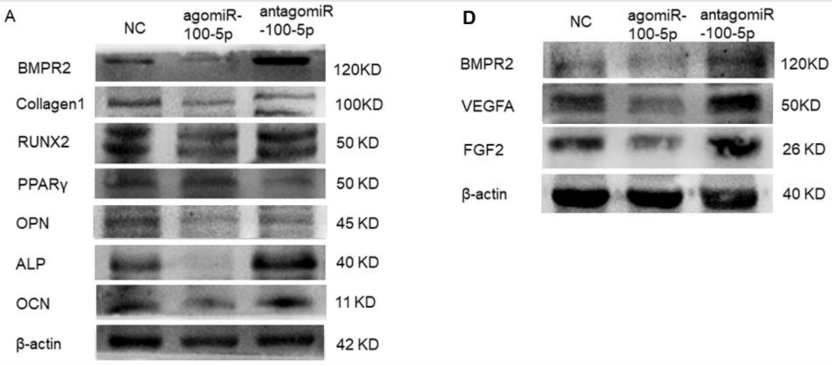

Figure6 A and D repeat 1

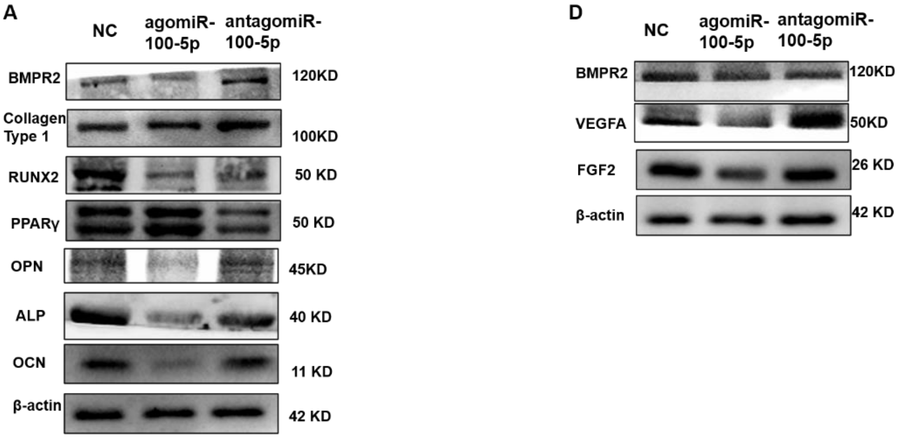

Figure6 A and D repeat 2

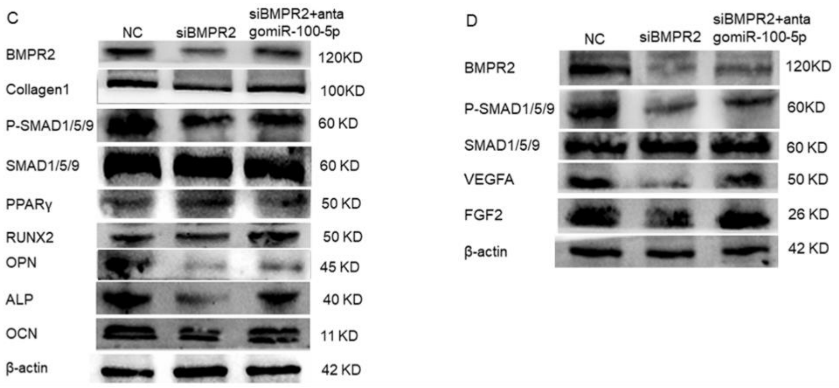

Figure7 C and D repeat 1

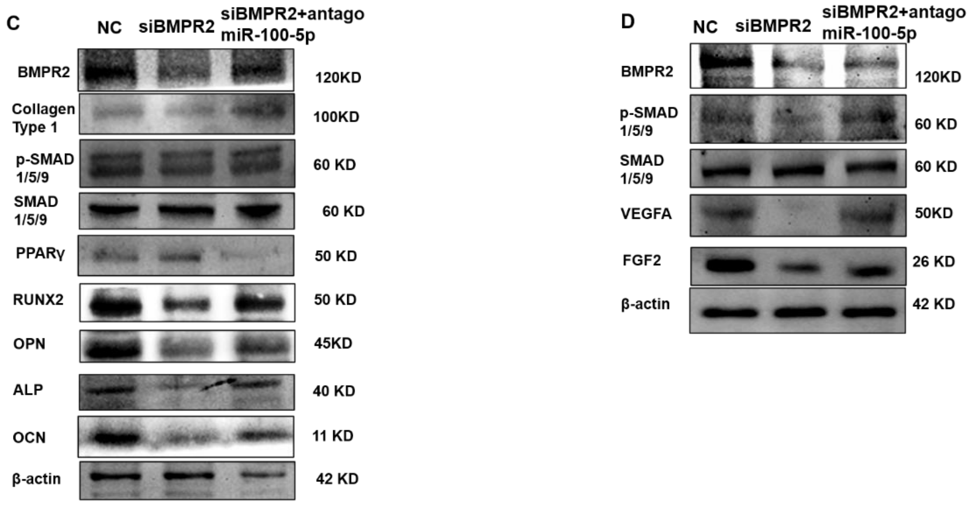

Figure7 C and D repeat 2

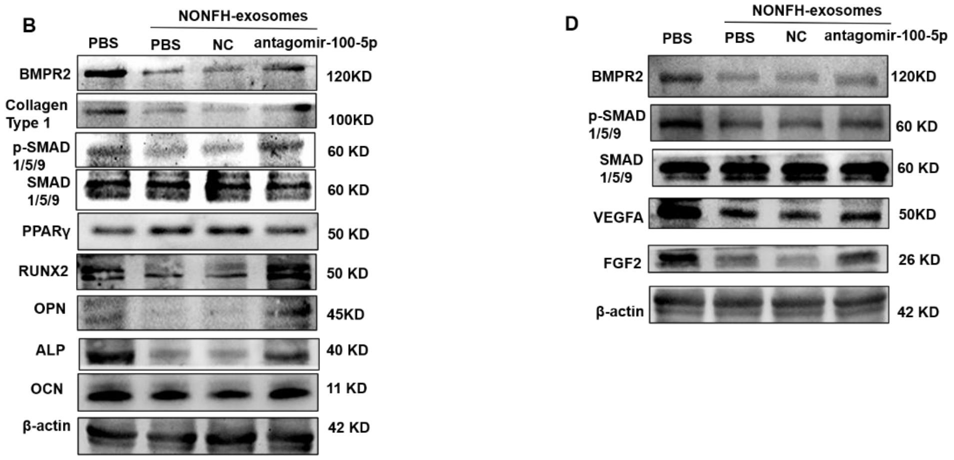

Figure8 B and D repeat 1

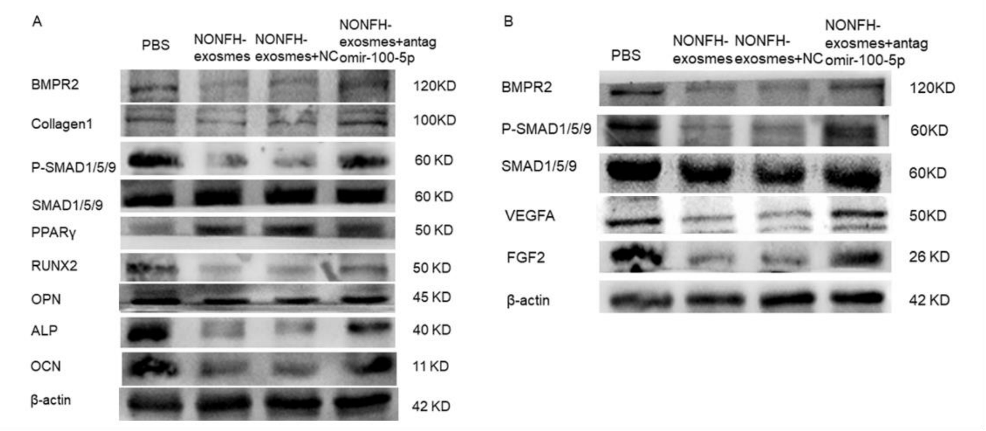

Figure8 B and D repeat 2
